# Supplementary material for: Bioactivity descriptors for uncharacterized chemical compounds
Source: Nat Commun. 2021 Jun 24;12:3932. doi: 10.1038/s41467-021-24150-4 (PMC8225676; doi:10.1038/s41467-021-24150-4)
Supplement: Supplementary file 1 — Supplementary Information [file 41467_2021_24150_MOESM1_ESM.pdf]

Supplementary Figures and Tables

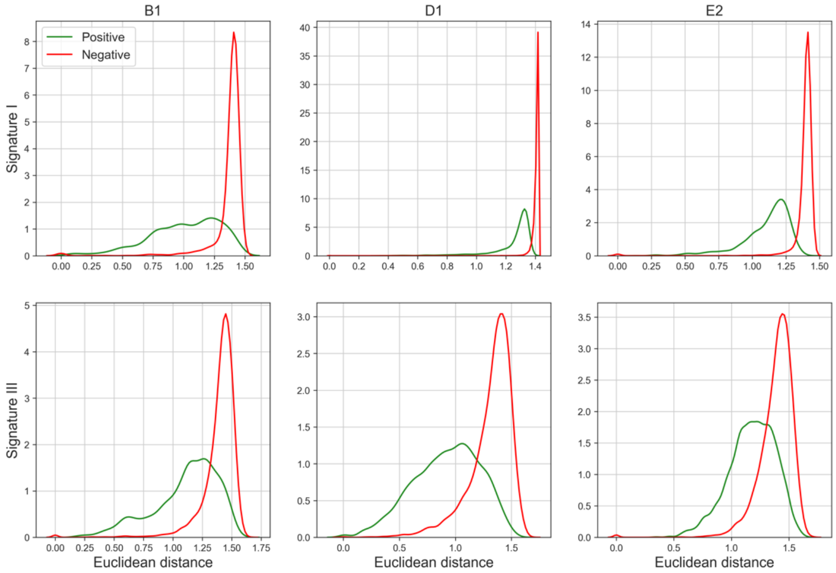

Supplementary Figure 1. Original and learnt triplet distances for three representative CC datasets, namely B1, D1 and E2. The upper row shows the anchor-positive (green) and anchor-negative (red) Euclidean distances observed in the signature type I space (i.e. experimental signatures). Positive samples are closer to the anchor than negative ones. Correspondingly, the bottom row shows the distances observed in the signature III space (i.e. SNN embedding). Only test-test comparisons, where none of the molecules were seen during training, are shown.

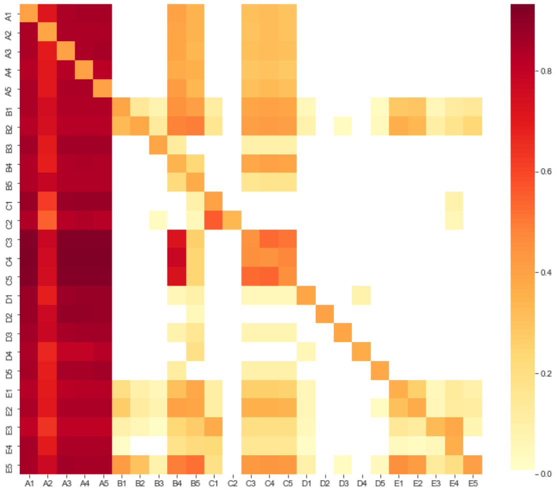

Supplementary Figure 2. Proportion of signatures kept by the signature-dropout strategy. Rows (i) represent the CC space for which the SNN is being trained, and columns (j) correspond to the signatures being sampled. Red indicates that j signatures were typically used to train the i signaturizer.

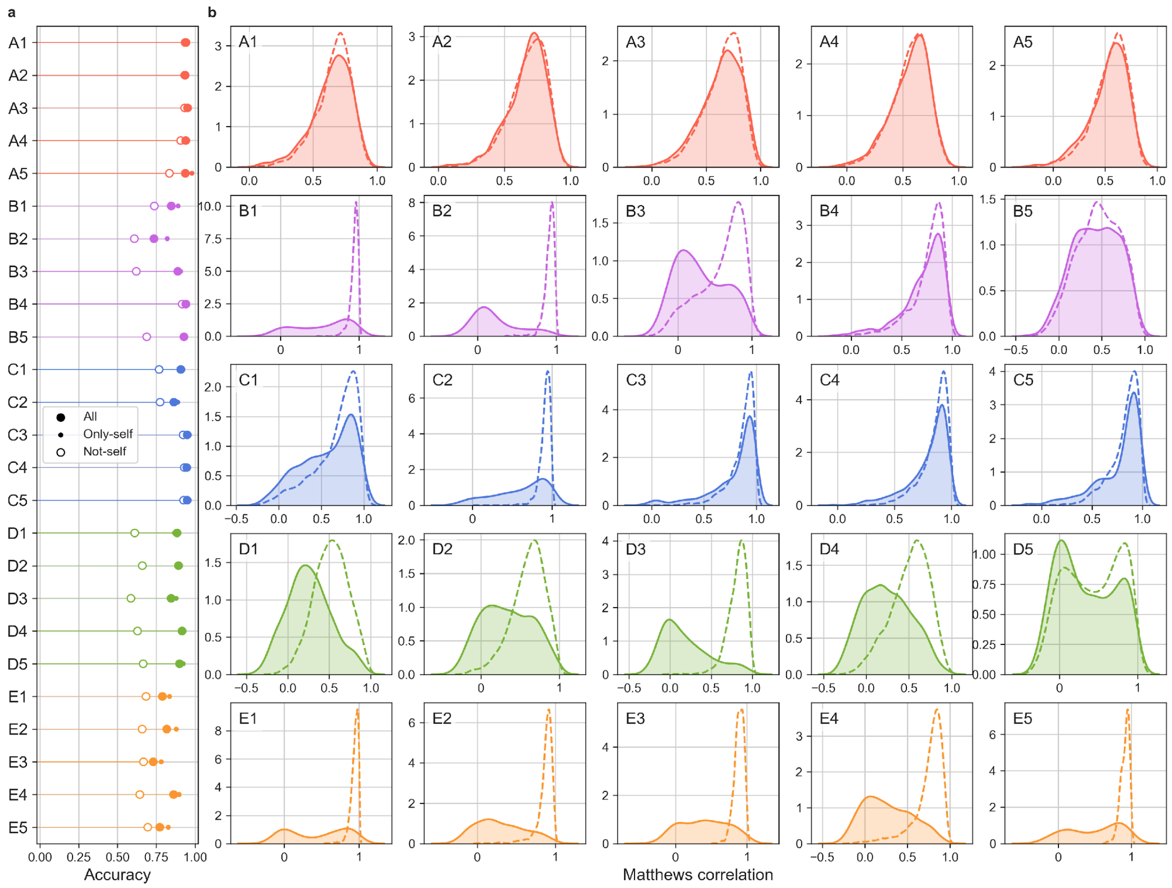

Supplementary Figure 3. Performance of the signaturizers. (**a**) Performance (measured as a triplet-resolving accuracy) of signatures produced using all data (including the space of interest, all), only the space of interest (only-self), and not using the space of interest (not-self). Related to Figure 1c. (**b**) MCC scores (predicted signature vs known signature) for train and test samples, depicted as dashed lines and filled shapes, respectively. Related to Figure 1d.

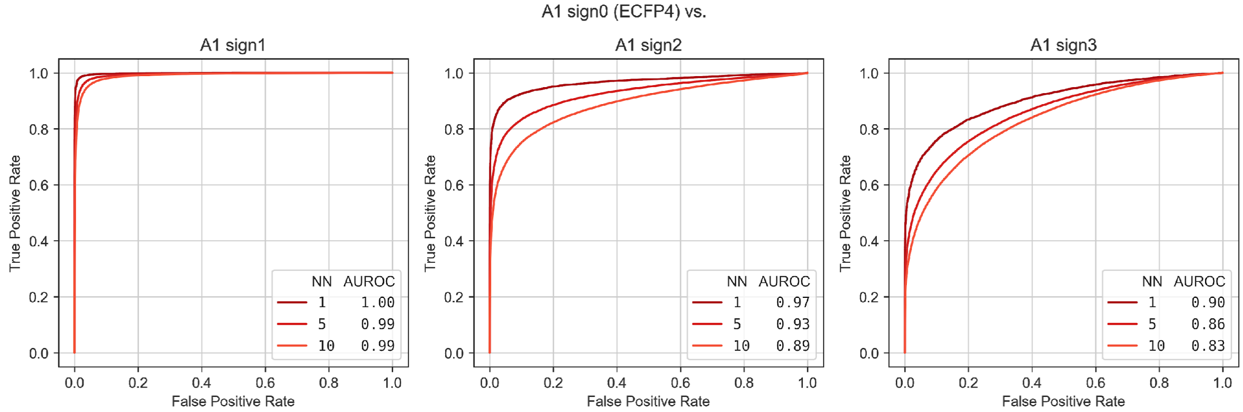

Supplementary Figure 4. AUROCs showing the capacity of chemistry A1 signatures (Sign1, Sign2 and Sign3) to recapitulate the 1, 5 and 10 nearest neighbors according to MFp descriptors for a sample of 10,000 randomly selected compounds.

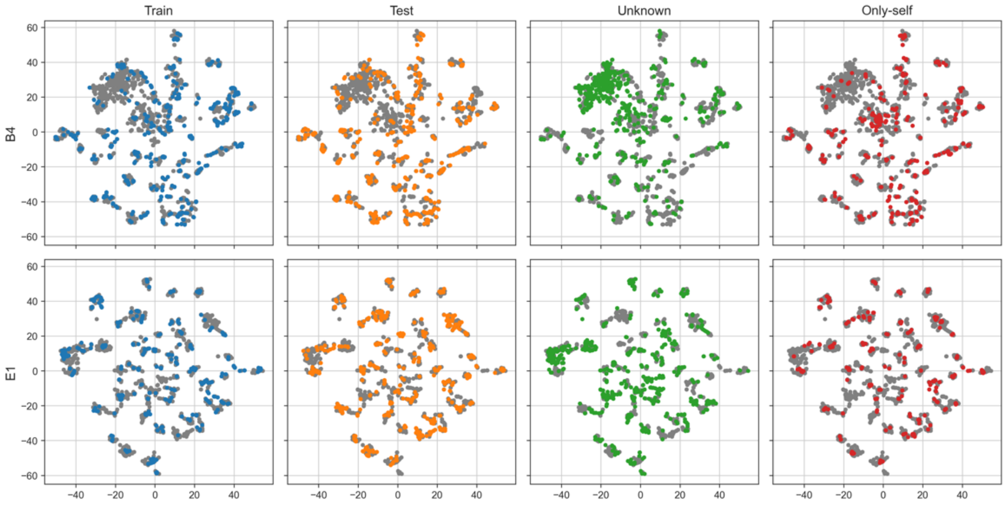

Supplementary Figure 5. t-SNE 2D projections for two exemplary datasets (B4 and E1). The first two columns correspond to molecules in the training and test sets. Unknown refers to signatures obtained for molecules with no available annotation in the space. Only-self shows predictions done taking only the B4/E1 space as input.

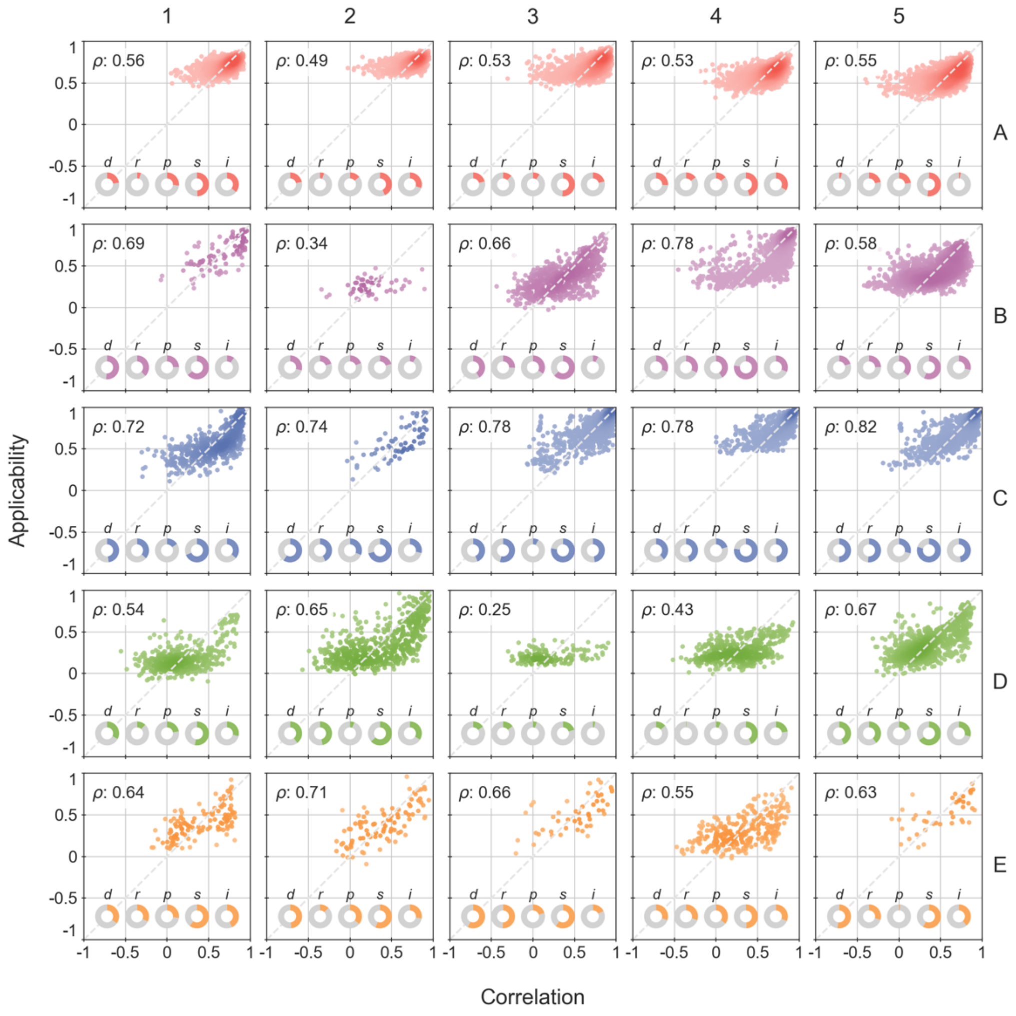

Supplementary Figure 6. Correlation between the applicability score (α) and the true-vs-predicted signature correlation of each bioactivity signature (top and right labels). The applicability score is determined by the linear combination of five factors, represented in the pie charts and abbreviated as follows; d: nearest-neighbor distance, r: robustness, p: prior (i.e. expected accuracy a priori), s: supervised distance, and i: intensity. The area covered by the pie chart corresponds to the coefficient of the linear combination to adjust an α score. Plots correspond to 80:20 train-test splits. Color intensity indicates the density ranging from low (light) to high (dark).

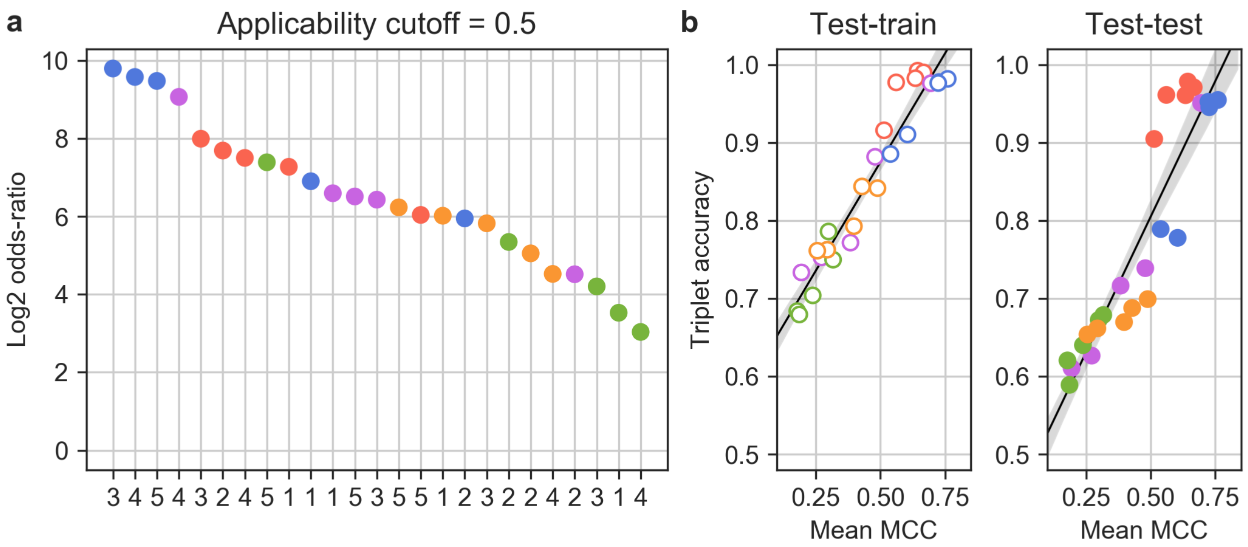

Supplementary Figure 7. (**a**) Enrichment of GSig 10-nearest-neighbors at an *α* cutoff of 0.5, measured as the log_2-odds ratio on a contingency table counting the number of neighbors common to S_i and GSig. High enrichments mean that, in the light of the global information available from the CC (i.e. GSig), similarities encountered for the S_i signature are relevant. The 25 CC categories are ranked by enrichment score. Color of the dot denotes CC level (A-E) and numbering indicates the sublevel (1-5). (**b**) Correlation between the two accuracy metrics (i.e. MCC and triplet accuracy) for train-train and test-test validations. The solid lines are simple linear regression models and the shaded area spans the 95% confidence interval.

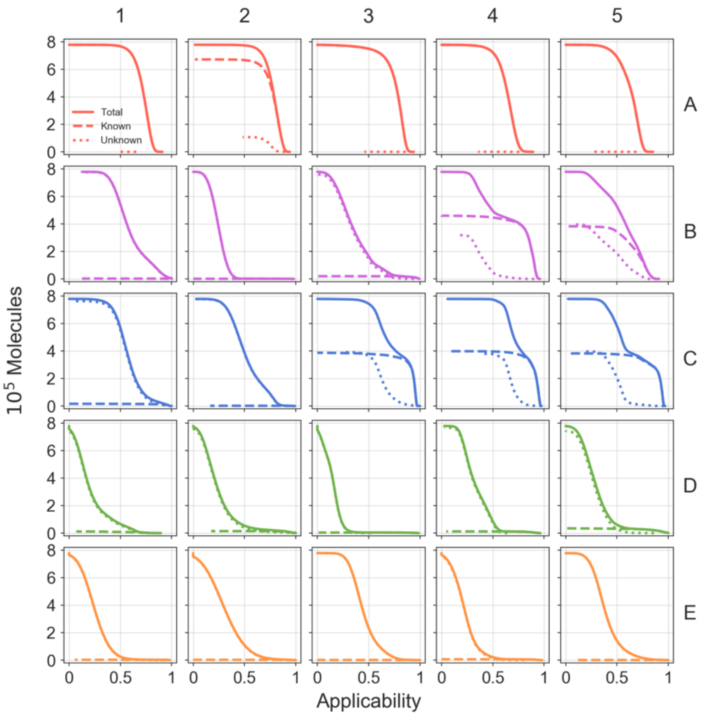

Supplementary Figure 8. Number of molecules at different applicability (α) cutoffs, obtained for the full universe of CC molecules (~800k) in each bioactivity signature (top and right labels). The dashed line indicates the number of molecules with experimental data in the corresponding CC space, while the dotted line indicates the number of molecules with no available data in the CC space. The solid line shows the total number of molecules. We can see that, for instance, with an α cutoff of 0.5 the number of molecules with reliable D1 signatures is 5-fold the number of molecules with experimental information.

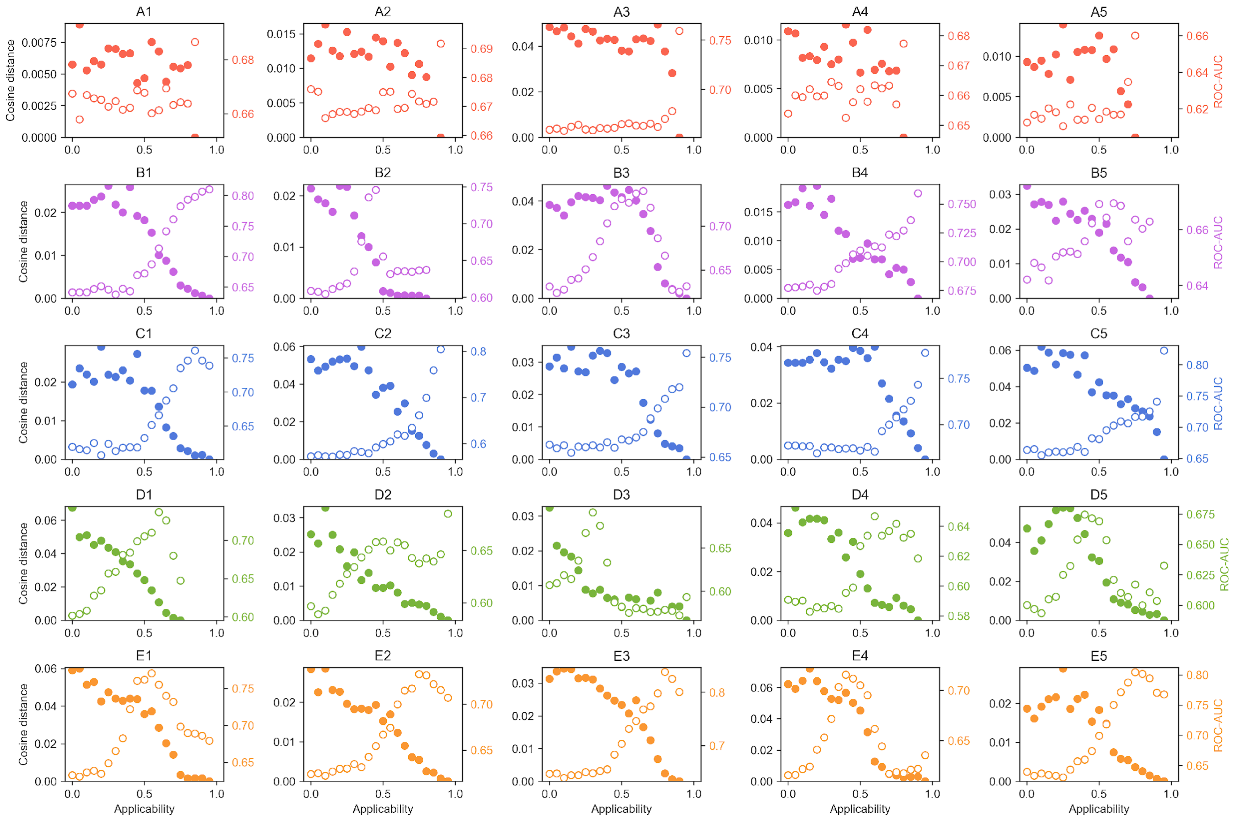

Supplementary Figure 9. Exploration of the α score. For each CC dataset S_i, we measure, at a given α cutoff, the capacity to recall 10-nearest-neighbors across CC spaces, similar to what is done in Figure 2c. The average ROC-AUC of S_i along $S_{1\text{-}25}$ is plotted as empty dots (right, colored axis). We consider the profile of ROC-AUCs obtained at the highest α to be the most genuine for the signature. We measure how the profile of ROC-AUCs diverges (cosine distance of the 25-dimensional ROC-AUC vector) as lower α values are taken. Larger distances indicate less purity in the signature-correlation profile (filled dots, left axis).

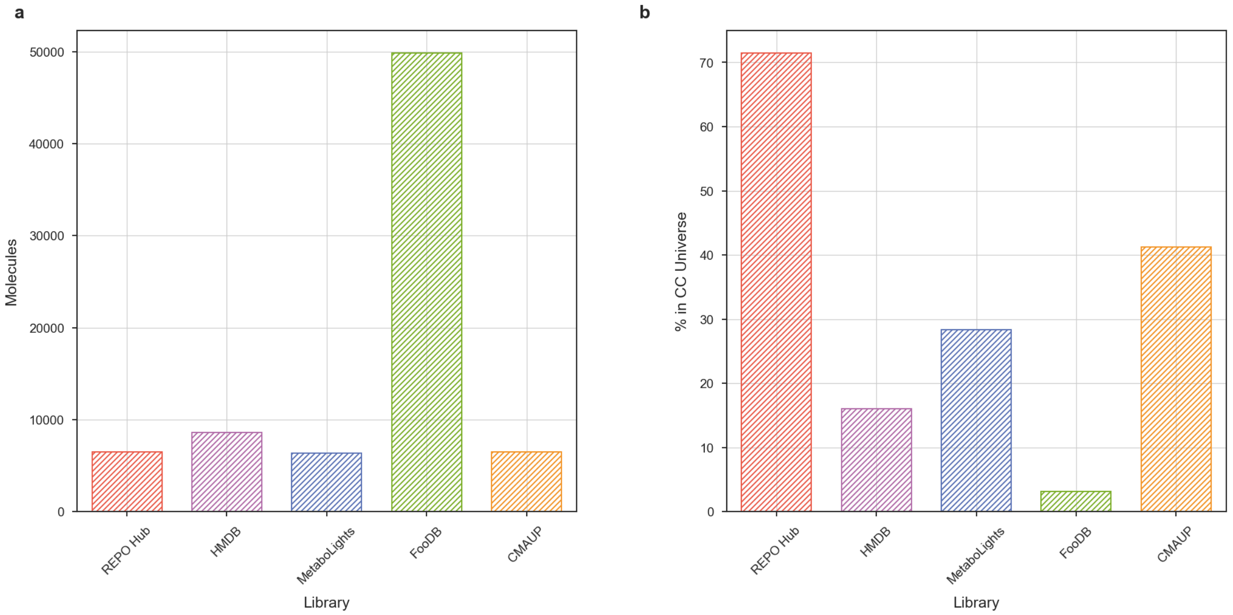

Supplementary Figure 10. Number of molecules (**a**) and CC coverage (**b**) of five selected compound collections with respect to the CC universe (~800k molecules).

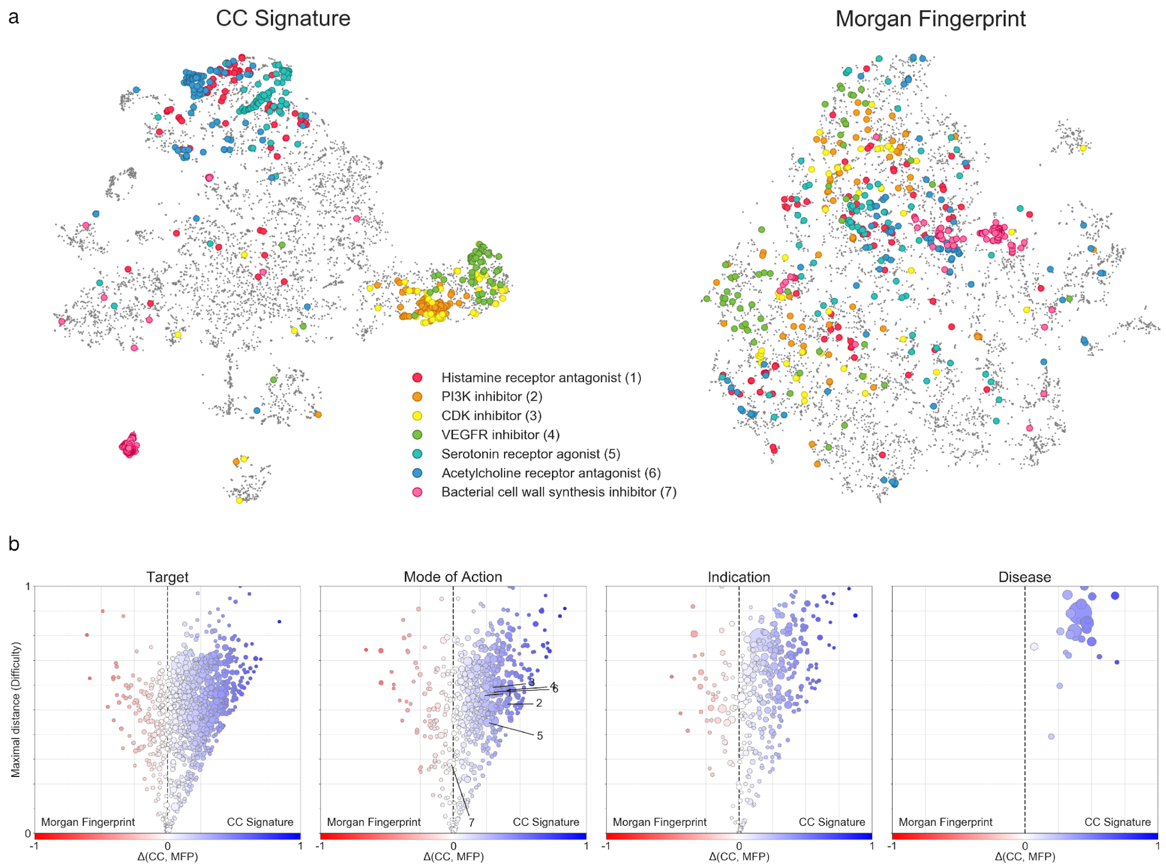

Supplementary Figure 11. Drug Repurposing Hub 2D projections. (**a**) t-SNE 2D projections based on GSigs (left) compared to Morgan fingerprint (MFp; 2048-bit, radius: 2) projections (right). Regions corresponding to certain MoAs are highlighted. (**b**) Level of clustering of the different annotations specified in the Drug Repurposing Hub, namely targets, MoA, indications and disease areas. Each dot corresponds to an annotation, and the size of the dot is proportional to the number of molecules. The average Euclidean distance in the 2D-projection between molecules with the same annotation is calculated, both for GSig- and MFp-based projections. For each annotation size, 100 randomly sampled points are drawn from the projection in order to scale the average distance measure. The x-axis measures the difference between GSig and MFp distances. Values close to 1 indicate that molecules of a certain annotation are well localized in the GSig projection and scattered in the MFp projection. Values close to -1 indicate the contrary. The red-to-blue color scale follows this axis. The y-axis is a measure of difficulty, i.e. the maximal inter-annotation distance observed between the GSig and MFp projections. Values close to one indicate that, in one of the projections the distance between molecules in the annotation is large (i.e. scattered points), while values close to 0 indicate that in both projections molecules with the same annotation are close-by. Thus, points in the upper-right corner are favorable to the CC projection, points in the mid-bottom region are well-grouped in both projections, and points in the upper-left corner are favorable to the MFp projection. The numbering in the MoA subplot relates to the legend in the top panel.

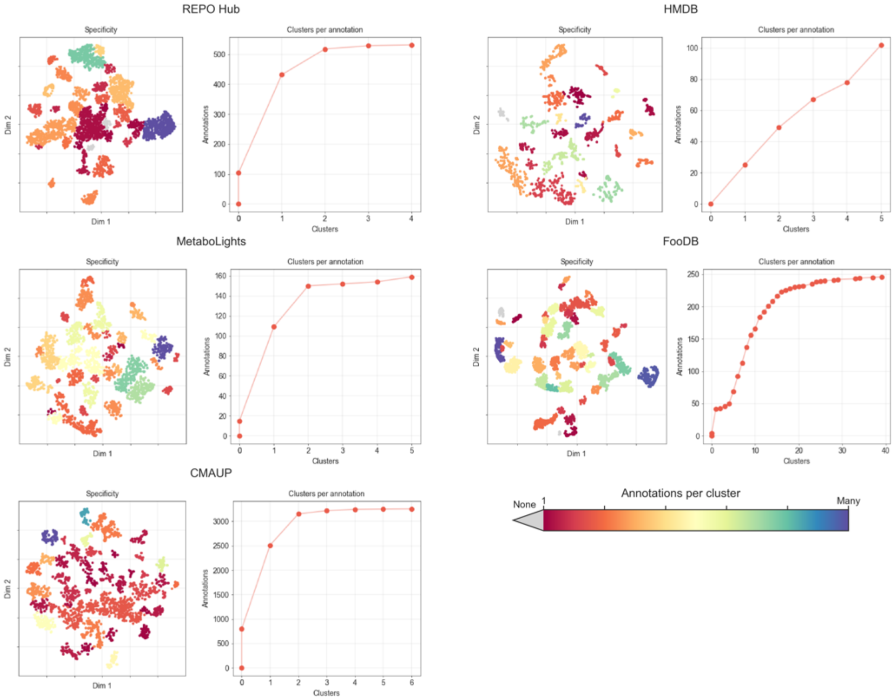

Supplementary Figure 12. Association analysis between clusters discovered in 2D projections and annotations available for the compounds. REPO Hub, HMDB, MetaboLights, FooDB and CMAUP datasets are analyzed separately. REPO Hub annotations: targets, MoAs, indications, disease areas. HMDB annotations: tissues, biofluids, biofunctions, cellular components and origin. MetaboLights annotations: organism name, group, genus, species, etc. FooDB: food group, subgroup and name. CMAUP: plant family and species. The DBSCAN clustering algorithm was used to identify clusters based on the (*x*,*y*)-coordinates of the 2D projection. Then, a one-sided Fisher's exact test was performed for each cluster-annotation pair, based on a contingency table counting the number of molecules in/out of the cluster and the number of molecules with/without annotations; P-values < 0.01 with an FDR < 0.1 and a log2 odds-ratio > 1.5 were considered to be significant. In the plots, the color of the projections denotes the number of annotations found to be statistically associated with each of the clusters. Blue regions are unspecific, in that they contain molecules belonging to multiple annotations. Red regions are more specific as they are associated with few annotations. Gray clusters are not enriched with any annotation. The cumulative plots show the number of clusters associated with each annotation. In REPO Hub, for instance, most annotations are associated with one or two clusters, whereas most FooDB annotations are found enriched in five clusters.

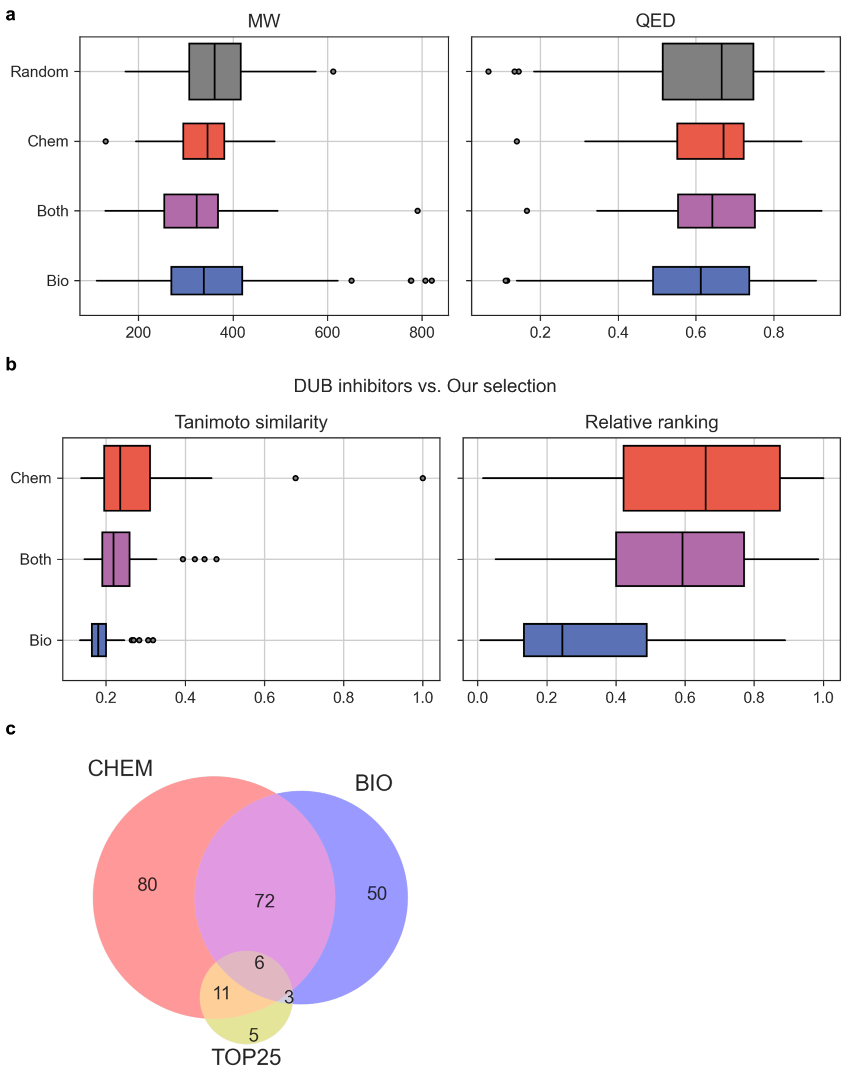

Supplementary Figure 13. (**a**) Molecular weight (MW) and quantitative estimate of drug-likeness (QED) of all the compounds experimentally tested for activity against Snail1, splitting them according to the queries that selected them (chemistry only in red, biological only in blue, both queries in lilac, and random in grey). The width of the box-plots is proportional to the number of molecules (n = 188, 91, 78, 53 from top to bottom), the box extends from the lower to upper quartile values of the data, with a line at the median, whiskers extend 1.5 times the IQR. (**b**) Tanimoto similarity and relative ranking of the 222 selected compounds against Snail1 compared to the closest known DUB inhibitor. The width of the barplots is proportional to the number of molecules (n = 91, 78, 53 from top to bottom), the box extends from the lower to upper quartile values of the data, with a line at the median, whiskers extend 1.5 times the IQR. (c) The Venn diagram shows the overlap between the 169 small molecules selected from the Chemical query, the 131 compounds selected from Biological queries and the Top25 active against Snail1, according to our dual-luciferase screen. Please, note that the Top25 active compounds also include two concentrations of the positive control (PR619) and three randomly selected compounds.

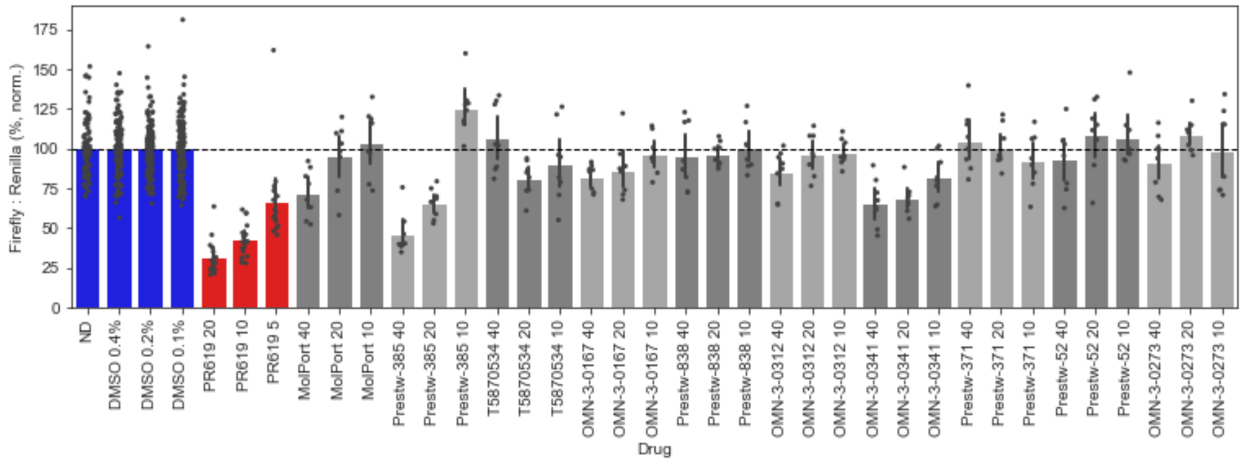

Supplementary Figure 14. Top10 activity compounds tested on the dual-luciferase assay with three doses (40, 20 and 10 μM). Five of the compounds, namely 1, 2, 4, 7, 9 show a dose-dependent inhibition of the Snail1 activity. The bar plot shows the average and standard deviation over two independent experiments.

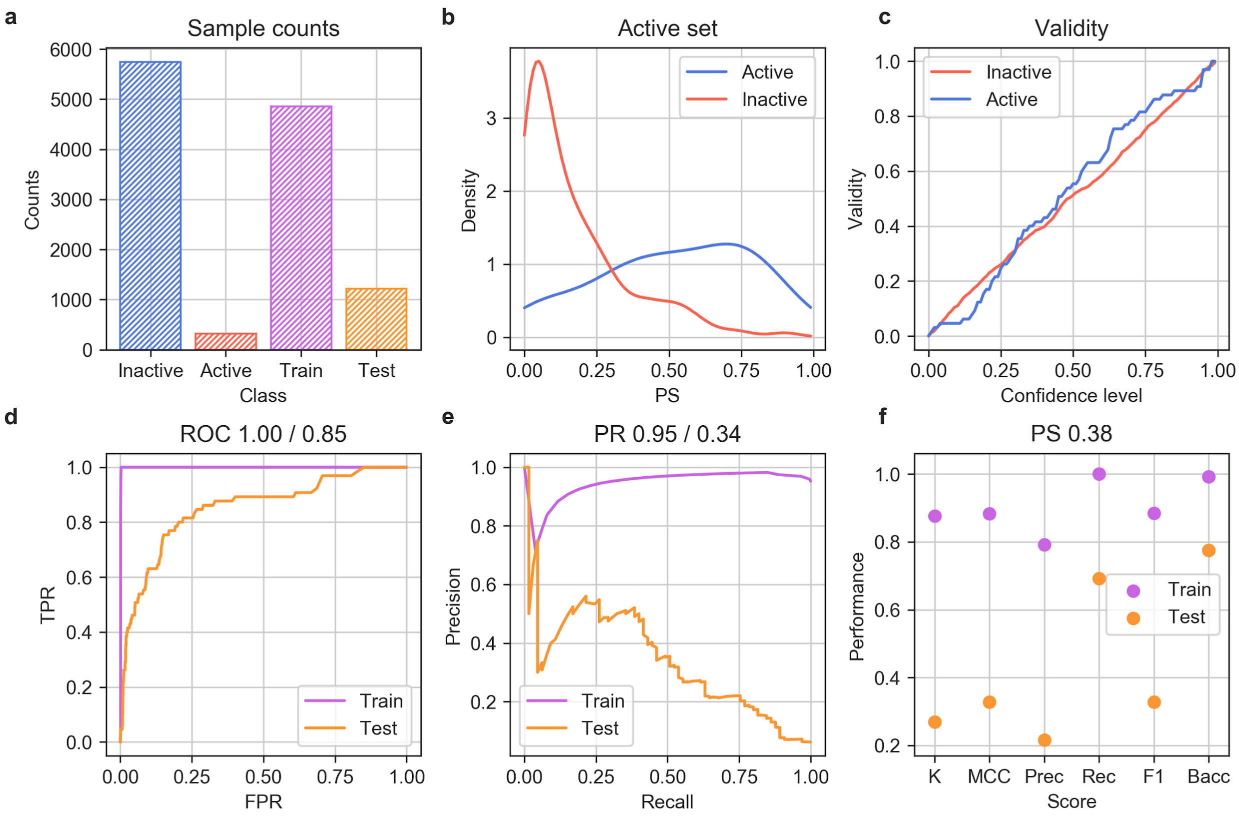

Supplementary Figure 15. Evaluation of the SR-HSE random forest classifier. (**a**) Number of active and inactive molecules, and proportion of molecules in the train and test sets. (**b**) Prediction score (PS) assigned to active (blue) and inactive (red) molecules in the test set. (**c**) Validity plot of the cross-conformal predictor. (**d**) Train and test ROC curves. (**e**) Train and test precision-recall curves. (**f**) Other classification scores (PS > 0.38). K: Cohen's kappa. Prec.: precision, Rec.: recall, Bacc: Balanced accuracy.

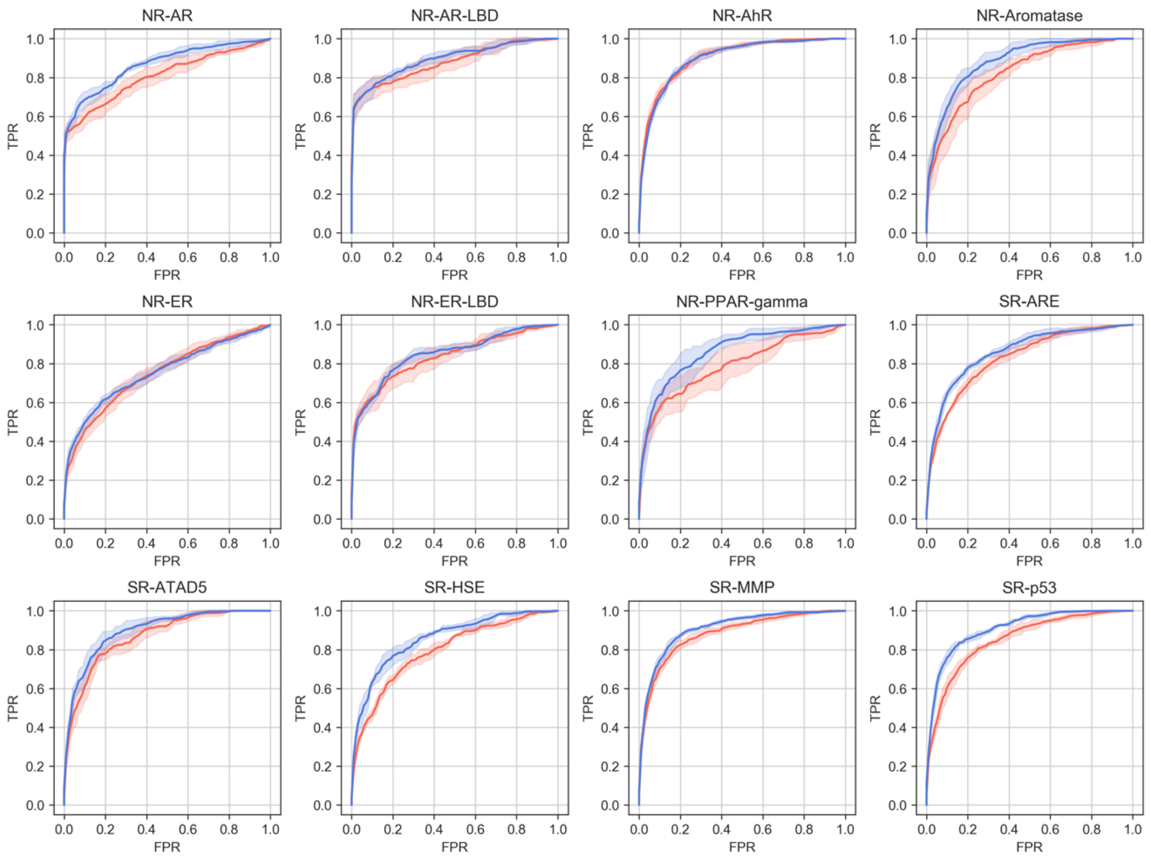

Supplementary Figure 16. ROC curves for the 12 Tox21 prediction tasks. CC- and MFp-based predictors are blue and red, respectively. Shaded areas span the standard deviation over five stratified train-test splits, the darker lines indicate the mean value.

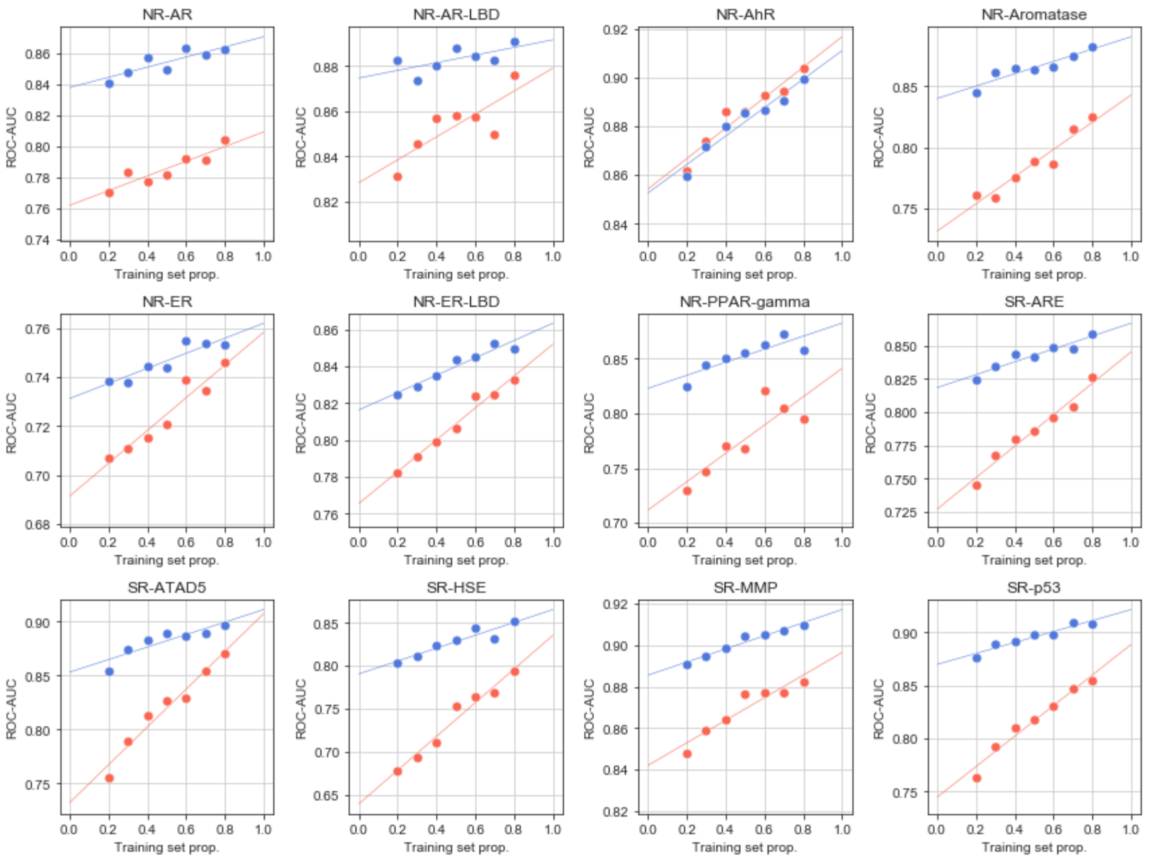

Supplementary Figure 17. Performance (ROC-AUC) of the Tox21 models at different training set sizes and using GSigs (blue) and MFps (red) as feature vectors.

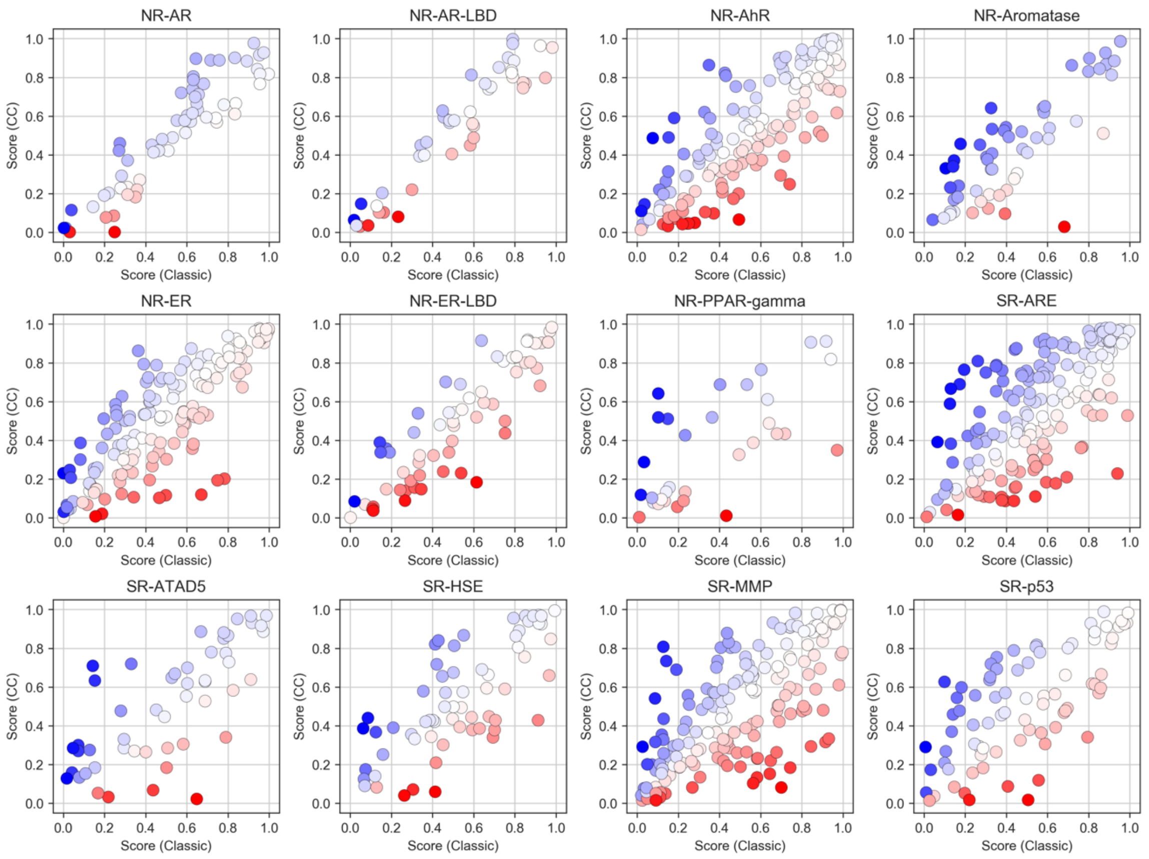

Supplementary Figure 18. Prediction scores assigned to each active molecule by the predictors at test-time in a 5-fold cross-validation (Tox21 benchmark dataset). The color scale denotes the relative difference between CC scores and classic (MFp) scores. Blue indicates a high score by the CC predictor and a low score by the MFp predictor. Red indicates the opposite.

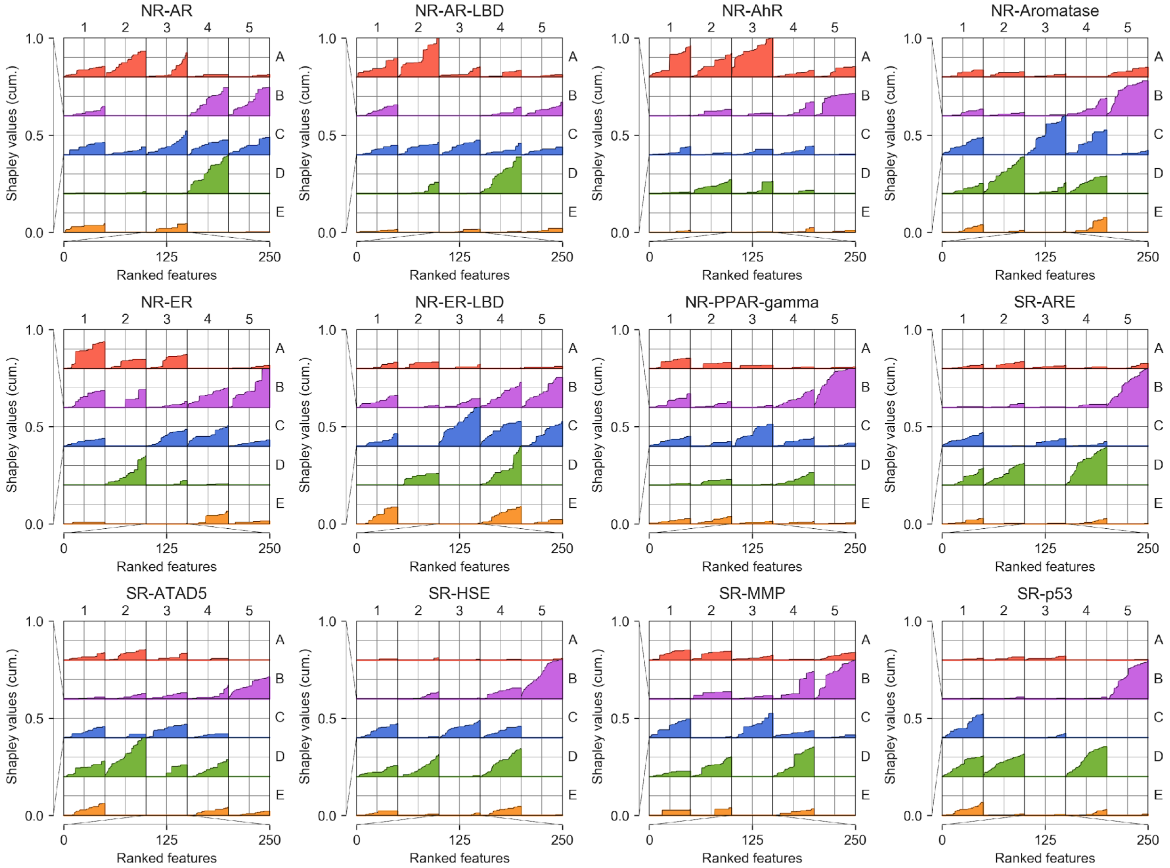

Supplementary Figure 19. Explanatory potential of CC categories across the Tox21 benchmark dataset. Top 250 features (ranked by average absolute Shapley values across samples) are summed up in the corresponding $S_{1\text{-}25}$ slots.

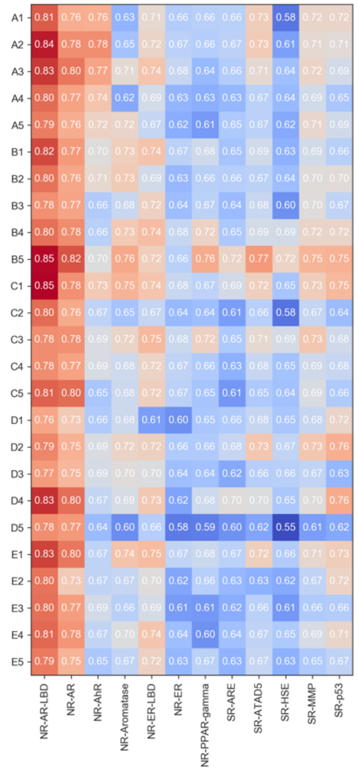

Supplementary Figure 20. Discriminative power (ROC-AUC) of SVCs based exclusively on 2D representations of the signatures. The analysis is done for each signature type (A1-E5) and the 12 Tox21 tasks. The color scale goes from blue to red indicating low to high discriminative power.

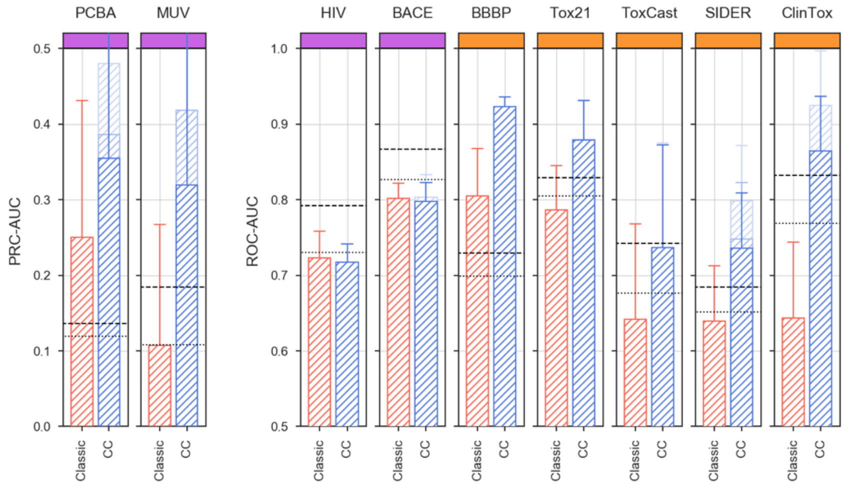

Supplementary Figure 21. MoleculeNet validation done exclusively with molecules having at least one bioactivity data point previously available in the CC. The plot relates to Figure 5g (see legend for details). We report Mean ± SD, n is defined in the original MoleculeNet publication.

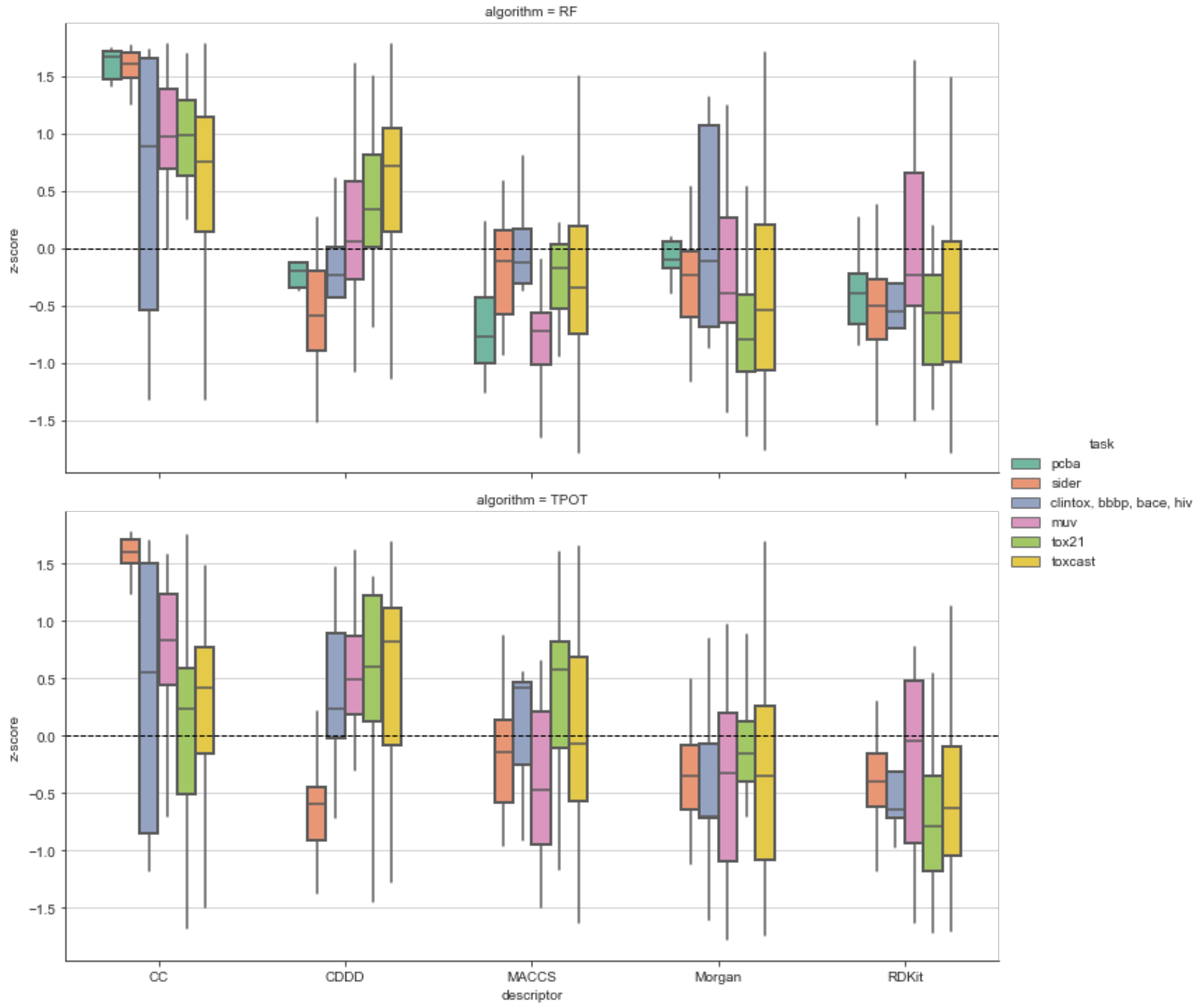

Supplementary Figure 22. Relative performance of CC descriptors across the MoleculeNet. A z-score is calculated for each task based on the performance scores for that particular task across descriptors. The box-plot extends from the lower to upper quartile values of the data, with a line at the median, whiskers extend 1.5 times the IQR. For computationally intensive tasks (e.g. PCBA) we subsampled 10 tasks randomly. Please note we use a fully stacked CC signature (25x128=3,200 dimensions). The prediction tasks are sorted according to the median of the CC performance. The top panel reports the result of a random forest classifier (n = 10, 27, 5, 17, 12, 404 following the legend order) and the bottom panel reports results obtained using TPOT classifiers (n = -, 27, 5, 17, 12, 70 following the legend order) .

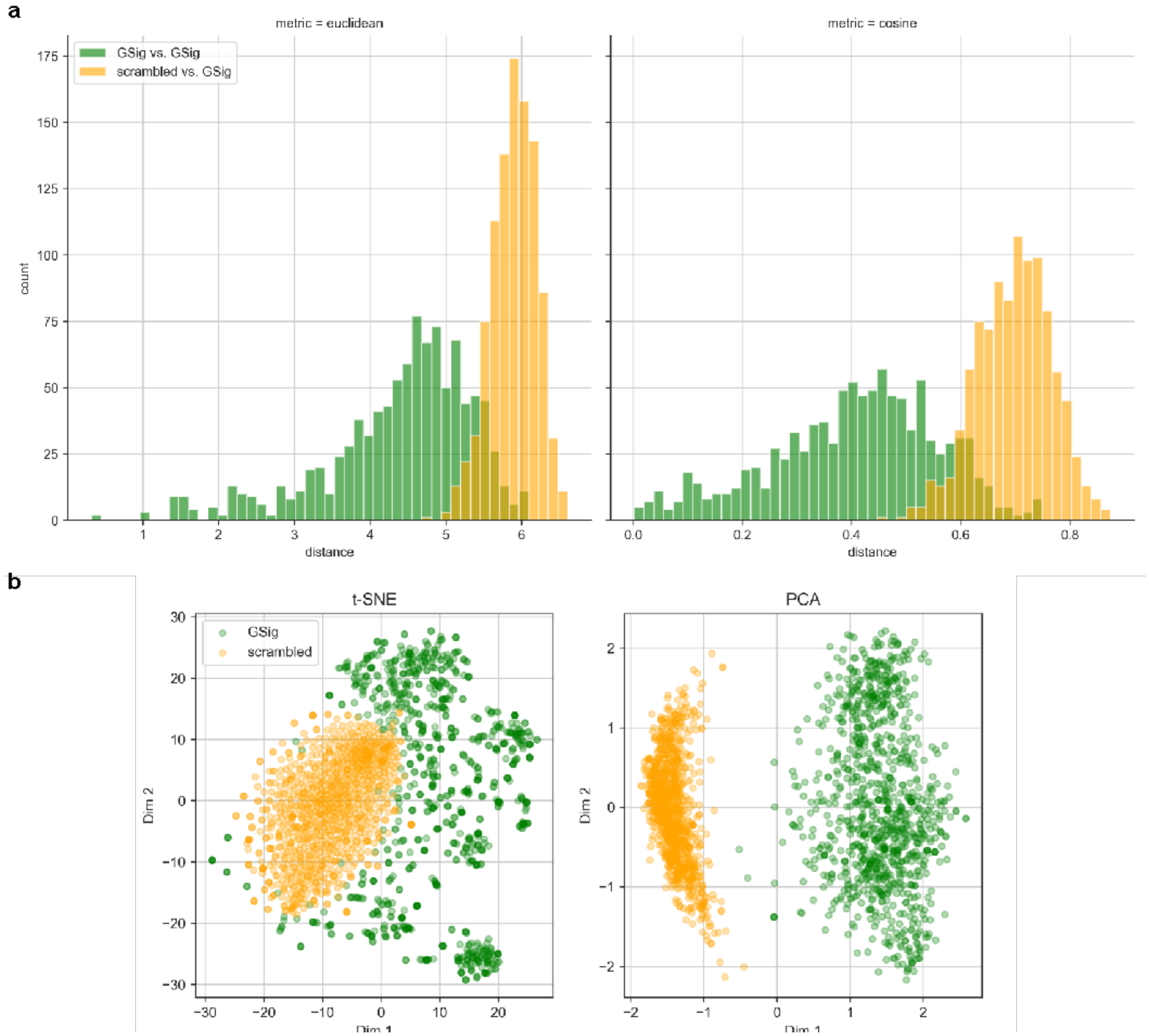

Supplementary Figure 23. (**a**) Distribution of all-against-all Euclidean and cosine distances between GSigs (green) and between scrambled and GSigs (orange). (**b**) Distribution of the closest Euclidean and cosine distances between pairs of molecules within the GSig set and scrambled-GSig. The plots show how there are no significant similarities between scrambles and global signatures, while GSigs can indeed detect similarities between small molecules (i.e. short distances between GSigs).

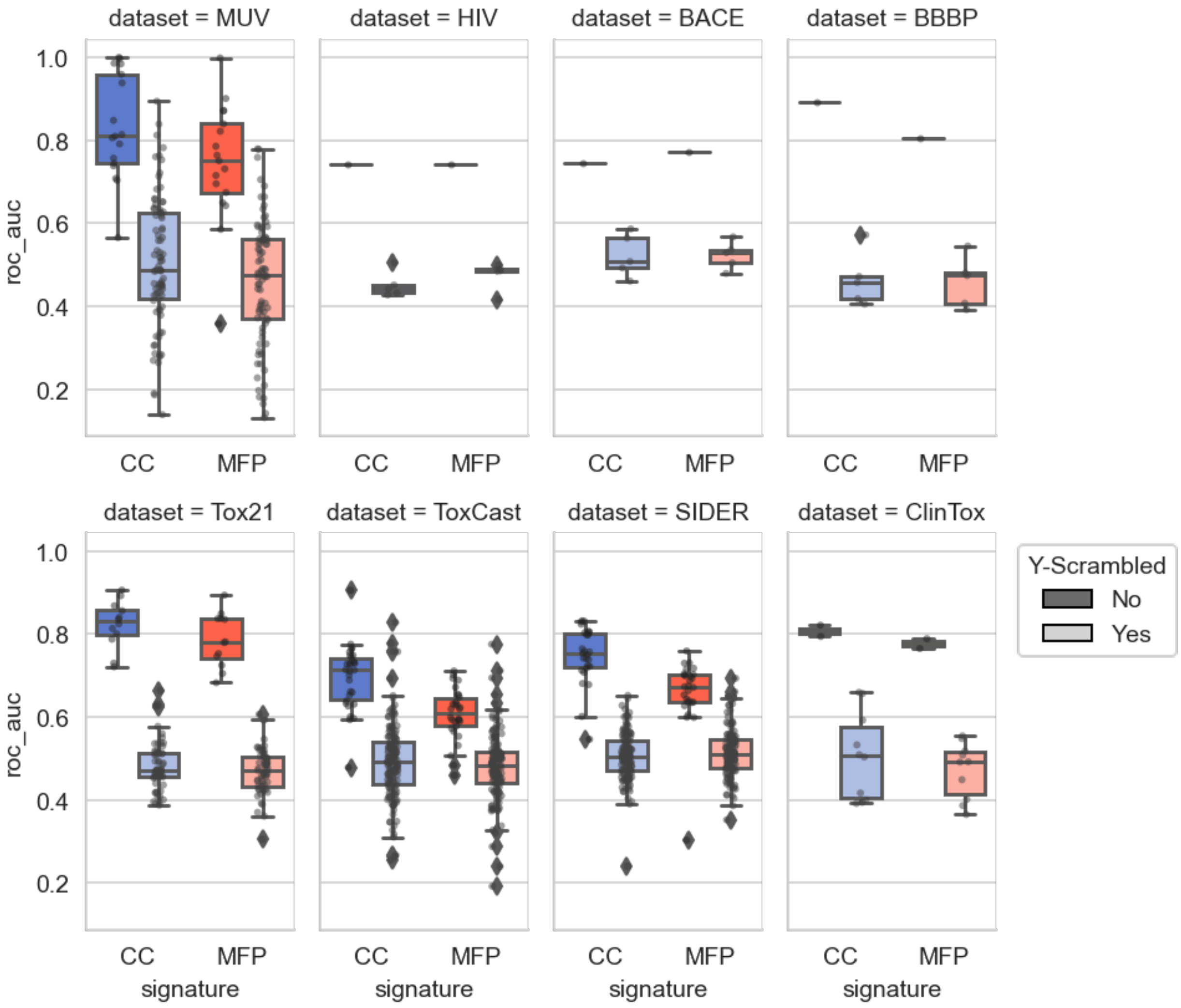

Supplementary Figure 24. Y-scrambling experiment on MoleculeNet task. We performed a simple logistic regression using both GSig (CC in shades of blue) signature and ECP4 (MFP in shades of red) descriptors. The box in dark shades corresponds to normal classification on unscrambled Y data, while the light shades correspond to 5 repetition of scrambling Y data.

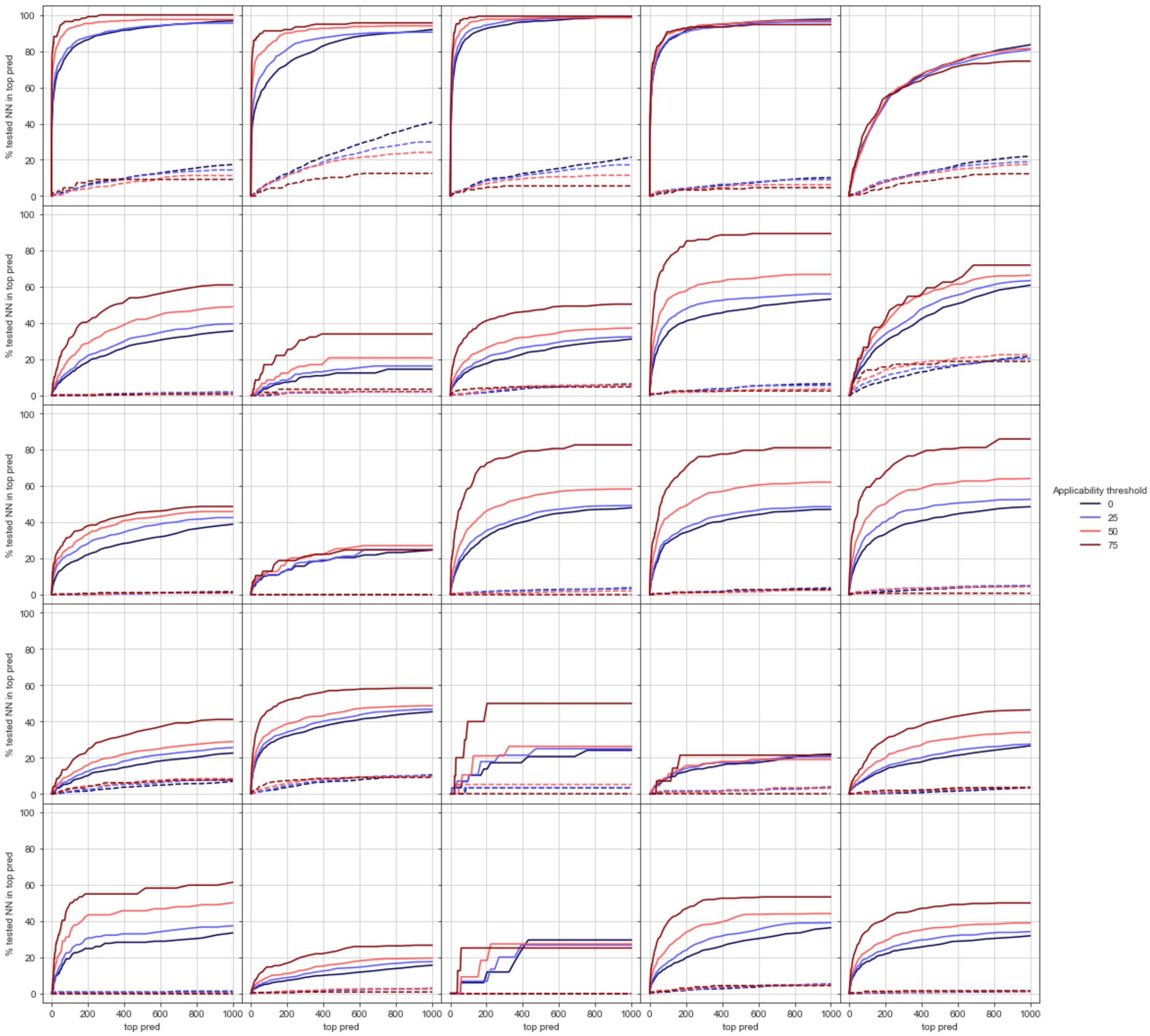

Supplementary Figure 25. Time-series validation of the signaturizers in all bioactivity spaces. The plots show the ability of the signaturizers to identify similar molecules to compounds not yet annotated when the signaturizers were derived, and we compare them to a random expectation. Despite the limited number of new molecules with experimental information in some of the spaces, we can see that the signaturizers derived from previous versions can identify similar molecules for a significant fraction of the new compounds, and this increases when we use more stringent applicability thresholds.

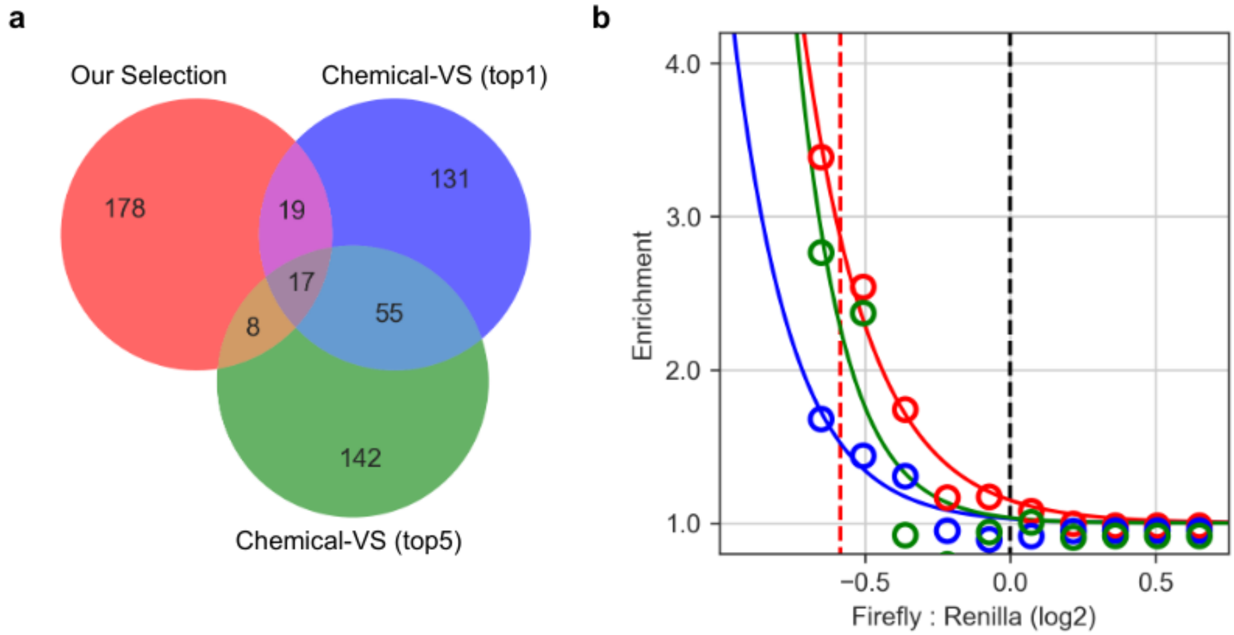

Supplementary Figure 26. (**a**) Venn diagram showing the overlap between 222 compounds selected from the IRB+Prestwick libraries (>17k molecules) using chemical checker signatures (our selection) and ECPF4-based strategies (Chemical-VS). (**b**) Fold enrichment of compounds selected by Signature-based (red), average distance to the closest 5 DUB inhibitors (Top5; green) and closest distance to any DUB inhibitor (Top1; blue) queries with respect to random picks, based on their capacity to modulate Snail1 levels (Firefly:Renilla assay).

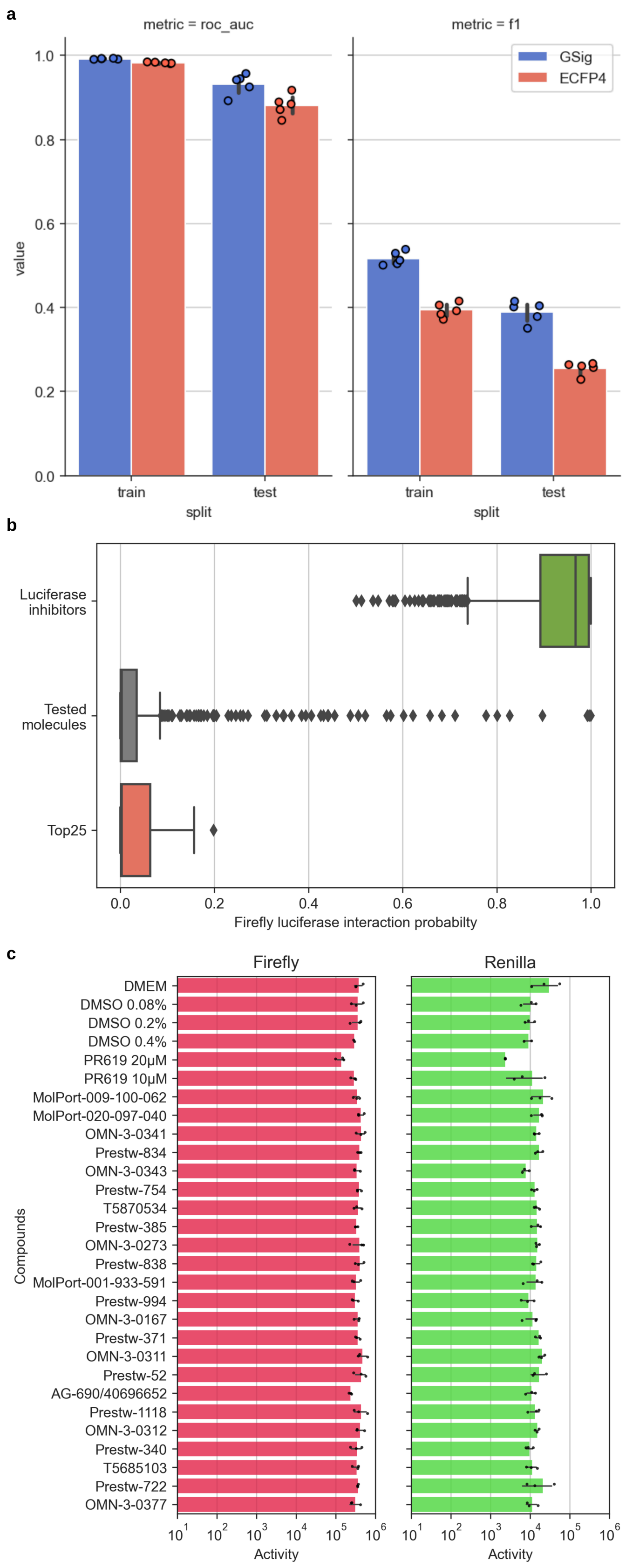

Supplementary Figure 27. (**a**) Performance of the logistic regression classifier trained to identify compounds with potential luciferase activity measured as the area under the ROC (left) and F1 (right) metrics when using Gsig and MFp molecular descriptors. (**b**) Results of the logistic regression classifier to identify luciferase activity on the set of known luciferase inhibitors (green), tested Snail1 modulation candidates (gray) and experimentally identified actives (Top25; red). As it can be seen, according to the classifier, the probability of any of the active molecules to interfere with luciferase activity is very small. The box-plot extends from the lower to upper quartile values of the data, with a line at the median, whiskers extend 1.5 times the IQR (n = 1577, 444, 25). (**c**) Results of the effect of the Top25 compounds on MDA-MB-231 cells expressing constitutively the Firefly and the Renilla Luciferases under the control of CMV and TK promoters, respectively. The bar plots show the mean ± SD results of two experiments done in triplicate.

Supplementary Table 1. MoleculeNet classification tasks. Removed column indicates the CC spaces that are not used to make predictions. The parenthesis denotes the most aggressive removal.

Category	*Name*	*Molecules*	*Tasks*	*Split*	*Metric*	*Removed*
Biophysics	PCBA	437,929	128	Random	PR-AUC	B5, (B4, C3, C4, C5)
	MUV	93,087	17	Random	PR-AUC	B4, (B5, C3, C4, C5)
	HIV	41,127	1	Scaffold	ROC-AUC	-
	BACE	1,513	1	Scaffold	ROC-AUC	(B4, B5, C3, C4, C5)
Physiology	BBBP	2,039	1	Scaffold	ROC-AUC	-
	Tox21	7,831	12	Random	ROC-AUC	(E4)
	ToxCast	8,575	612	Random	ROC-AUC	(E4)
	SIDER	1,427	27	Random	ROC-AUC	E3, (E4)
	ClinTox	1,478	2	Random	ROC-AUC	E1, E2, E3, E4, E5
