# Supplementary material for: Bioactivity descriptors for uncharacterized chemical compounds
Source: Nat Commun. 2021 Jun 24;12:3932. doi: 10.1038/s41467-021-24150-4 (PMC8225676; doi:10.1038/s41467-021-24150-4)
Supplement: Supplementary file 3 — Description of Additional Supplementary Files [file 41467_2021_24150_MOESM3_ESM.pdf]

**Description of Additional Supplementary Files**

File Name: Supplementary Data 1
Description: Library enrichment for activity against Snail1. This dataset contains the selected compounds from the IRB and PWCK libraries for their possible activity against Snail1, and the information we used to perform these queries (Methods). We indicate and prioritize (1) known DUB inhibitors, listing their known targets, (2) DUBs according to the results of the siRNA-DUB/Snail1 screening assay and (3) public transcriptional signatures related with the Snail1 activation pathway. We report the score of each molecule in the inspected queries (e.g. similarity to known DUB inhibitors, reversion of Snail1 transcriptional signatures, belonging to the TGFβ pathway, etc.). We provide information for all compounds selected in chemical and biological queries, as well as the randomly selected ones.
